# Hypoblast Formation in Bovine Embryos Does Not Depend on NANOG

**DOI:** 10.3390/cells10092232

**Published:** 2021-08-28

**Authors:** Claudia Springer, Valeri Zakhartchenko, Eckhard Wolf, Kilian Simmet

**Affiliations:** 1 Institute of Molecular Animal Breeding and Biotechnology, Gene Center and Department of Veterinary Sciences, Ludwig-Maximilians-Universität München, 85764 Oberschleissheim, Germany; c.springer@gen.vetmed.uni-muenchen.de (C.S.); v.zakhartchenko@gen.vetmed.uni-muenchen.de (V.Z.); ewolf@genzentrum.lmu.de (E.W.); 2 Center for Innovative Medical Models (CiMM), Ludwig-Maximilians-Universität München, 85764 Oberschleissheim, Germany; 3 Laboratory for Functional Genome Analysis (LAFUGA), Gene Center, Ludwig-Maximilians-Universität München, 81377 Munich, Germany

**Keywords:** NANOG, SOX17, bovine preimplantation development, MEK, second lineage differentiation

## Abstract

The role of the pluripotency factor NANOG during the second embryonic lineage differentiation has been studied extensively in mouse, although species-specific differences exist. To elucidate the role of NANOG in an alternative model organism, we knocked out *NANOG* in fibroblast cells and produced bovine *NANOG*-knockout (KO) embryos via somatic cell nuclear transfer (SCNT). At day 8, *NANOG*-KO blastocysts showed a decreased total cell number when compared to controls from SCNT (NT Ctrl). The pluripotency factors OCT4 and SOX2 as well as the hypoblast (HB) marker GATA6 were co-expressed in all cells of the inner cell mass (ICM) and, in contrast to mouse *Nanog*-KO, expression of the late HB marker SOX17 was still present. We blocked the MEK-pathway with a MEK 1/2 inhibitor, and control embryos showed an increase in NANOG positive cells, but SOX17 expressing HB precursor cells were still present. *NANOG*-KO together with MEK-inhibition was lethal before blastocyst stage, similarly to findings in mouse. Supplementation of exogenous FGF4 to *NANOG*-KO embryos did not change SOX17 expression in the ICM, unlike mouse *Nanog*-KO embryos, where missing SOX17 expression was completely rescued by FGF4. We conclude that NANOG mediated FGF/MEK signaling is not required for HB formation in the bovine embryo and that another—so far unknown—pathway regulates HB differentiation.

## 1. Introduction

Before implantation, mammalian embryos undergo two consecutive lineage specifications. First, outer and inner cells in the morula form the surrounding CDX2-expressing trophectoderm (TE) and the inner cell mass (ICM), respectively, during blastocyst development. Second, within the ICM, NANOG-expressing cells form the pluripotent epiblast (EPI) and exhibit a mutually exclusive expression pattern with differentiated cells expressing GATA6 and SOX17 from the primitive endoderm (PE) or hypoblast (HB) in bovine and human embryos. Consequently, three distinct cell lineages arise: the EPI, which will give rise to the embryo proper, the PE/HB, which will form the yolk sac, and the TE, responsible for extraembryonic tissues and implantation [1,2,3,4,5,6,7,8], reviewed in [9]. While these landmarks of preimplantation embryonic development are conserved between mammalian species, fundamental differences exist regarding the regulation of the second lineage segregation. Human and bovine *OCT4*-knockout (KO) embryos lose NANOG and maintain GATA6 expression, whereas mouse *Oct4*-KO embryos still express NANOG and fail to develop a PE [10,11,12,13]. Additionally, FGF4 signaling via the mitogen-activated protein kinase (MAPK) pathway, also called MEK pathway, has different roles in the regulation of the second lineage differentiation between species. It is known that in mouse, EPI precursor cells express FGF4, which via the FGF receptors 1/2 (FGFR) and the MEK-pathway induces and regulates the formation of the PE (reviewed in [14]). Inhibition of the MEK pathway or the FGFR in mouse embryos results in an ICM only expressing NANOG, while GATA6 expression is completely lost [15,16,17]. In both human and bovine embryos, inhibition upstream of MEK via FGFR inhibitors has no effect on EPI or HB formation [18]. In bovine embryos, MEK inhibition increases NANOG expression and reduces HB markers, but HB marker expression is still present. In human embryos, MEK inhibition has no effect, suggesting that bovine and human HB formation is partly or completely independent of this pathway, and that these species regulate the second lineage differentiation differently [18,19,20,21].

Recently, a dosage-dependent effect of the MEK-inhibitory compound PD0325901 (PD032) was found in bovine embryos. A concentration of 2.5 µM eliminated expression of the later HB-marker SOX17 completely [22], while previous studies observed maintenance of the early marker GATA6 at concentrations of 0.5 and 1 µM [18,20], challenging the hypothesis of a partly MEK-independent HB formation in bovine.

Supplementing exogenous FGF4 from morula to blastocyst stage leads to ubiquitous GATA6 expression in mouse, bovine, pig and rabbit embryos (reviewed in [23]), revealing a non-cell-autonomous role for FGF4.

NANOG is a member of the homeobox family of DNA binding transcription factors that is known to maintain the pluripotency of embryonic stem cells (ESCs) together with OCT4 and SOX2 [6,24]. In mouse, *Nanog*-KO does not affect the formation of the blastocyst, but during the second lineage differentiation, the EPI lineage fails. Thereby, ubiquitous expression of the early PE marker GATA6 within the ICM was reported, whereas the late PE markers SOX17 and GATA4 were lost [1,2,25]. SOX17 expression in mouse *Nanog*-KO embryos is rescued by supplementing exogenous FGF4, confirming the crucial role of FGF4 expressed by EPI cells for PE differentiation [2]. Mouse *Nanog*-KO embryos and ESCs lose viability in the presence of MEK inhibitors, resulting in early cell death [2,26]. So far, the phenotype of *NANOG*-KO in human embryos or ESCs has not been investigated, but a *NANOG*-knockdown experiment in human ESCs showed that, while NANOG represses embryonic ectoderm differentiation, it does not influence the expression of OCT4 or SOX2 [27]. In bovine embryos, NANOG is first expressed at the morula stage and becomes specific to EPI precursor cells in the ICM at day 7 [18,28,29]. Only recently, a *NANOG*-KO via zygote injection was first described in bovine, displaying pan-ICM GATA6 expression and reduced transcript levels for the pluripotency factors SOX2 and H2AFZ [30].

In the present study, we addressed the role of NANOG in bovine preimplantation embryos using a reverse genetics approach. After induction of a *NANOG* frameshift mutation in fibroblasts and production of embryos via somatic cell nuclear transfer (SCNT), we characterized the *NANOG*-KO phenotype by immunofluorescence staining of day 8 blastocysts for markers of EPI and HB precursor cells. We further addressed the roles of FGF4 and the MEK pathway by treating *NANOG*-KO embryos with exogenous FGF4 and with an inhibitor of the MEK pathway, respectively, revealing new insights into the second lineage differentiation in bovine embryos.

## 2. Materials and Methods

### 2.1. CRISPR/Cas9-Mediated KO of NANOG in Adult Fibroblasts

We induced a frameshift mutation in a non-homologous end joining approach with an sgRNA (5′-CTCTCCTCTTCCCTCCTCCA-3′) designed by Synthego software (V2.0) using ENSBTAT00000027863 (*NANOG*) as reference gene (design.synthego.com, accessed on 15 July 2021). The sgRNA targeting exon 2 of *NANOG* was cloned into pSpCas9(BB)-2A-Puro (PX459) V2.0, a gift from Feng Zhang [31]. All experiments are based on a cell line with origin in bovine adult ear fibroblast cells that were isolated in the laboratory in the Chair for Molecular Animal Breeding and Biotechnology, Ludwig-Maximilians-Universität München, 85764 Oberschleissheim, Germany. Bovine fibroblasts were transfected with the Nucleofector device (Lonza; Basel, Switzerland) according to the manufacturer’s instructions. After selection with 2 µg/mL puromycin (Sigma-Aldrich; St. Louis, MO, USA) for 48 h, we produced single-cell clones as described previously [32]. After PCR amplification with primers 5′-GGAAGGGATTCCTGAAATGAG-3′ (forward) and 5′-GTGGGATCTTAGTTGCGACAT-3′ (reverse), gene editing-induced modifications in the *NANOG* alleles and naturally occurring SNPs were examined by Sanger sequencing using the primers 5′-AAGGTCTGGGTTGCAATAGG-3′ (forward) and 5′-CCACCAGGGAAATCCCTTATTT-3′ (reverse). All primers were synthesized by Biomers.net (Ulm, Germany; accessed on 15 July 2021).

### 2.2. Production and Analysis of SCNT and IVP Embryos

SCNT and in vitro production (IVP) procedures were performed as described previously [33]. Briefly, bovine ovaries were collected at a slaughterhouse, and retrieved cumulus-oocyte-complexes were matured in vitro. After SCNT or fertilization of the oocytes (day 0), fused complexes and presumptive zygotes were cultivated in synthetic oviductal fluid including Basal Medium Eagle’s amino acids solution (BME, Sigma-Aldrich), Minimum Essential Medium (MEM, Sigma-Aldrich) and 5% estrous cow serum (OCS) from day 0 or day 1 up to day 8 for SCNT or IVP zygotes, respectively. After 8 days of culture, the zona pellucida was removed enzymatically using Pronase (Merck Millipore; Burlington, MA, USA), and embryos were fixed in a solution containing 2% paraformaldehyde [34].

### 2.3. Modulation of Signaling Pathways

Growth factors or inhibitors were supplemented from day 5 morula stage until day 8 blastocyst stage. The MEK1 and MEK2 inhibitor PD032 (Tocris; Bristol, UK) was used at 0.5 or 2.5 µM, and controls were cultured in equal amounts of DMSO (Sigma-Aldrich). 1 µg/mL human recombinant FGF4 (R&D Systems; Minneapolis, MN, USA) was added to synthetic oviductal fluid with 1 µg/mL heparin (Sigma-Aldrich).

### 2.4. Immunofluorescence Staining and Confocal Laser Scanning Microscopy

Before staining, embryos were incubated for 1 h at room temperature in a blocking solution containing 0.5% Triton X-100 (Sigma-Aldrich) and 5% donkey serum (Jackson ImmunoResearch; West Grove, PA, USA) or fetal calf serum (Thermo Fisher Scientific; Waltham, MA, USA) or both sera sequentially, depending on the species origin of the secondary antibodies. Double staining for either NANOG/GATA6, NANOG/SOX17, SOX17/SOX2, OCT4/SOX2 or GATA6/CDX2 was achieved by incubation overnight at 4 °C in primary antibody solution and transfer to secondary antibody solution at 37 °C for 1 h after washing 3 times. The antibodies used and the applied dilutions are presented in Supplementary Table S1. Labeled embryos were mounted in Vectashield mounting medium containing 4′,6-diamidino-2-phenylindole (DAPI, Vector Laboratories; Burlingame, CA, USA) in a manner that conserved the 3D structure of the specimen [35]. Z-stacks of optical sections with an interval of 1.2 µm were recorded using an LSM710 Axio Observer confocal laser scanning microscope (CLSM; Zeiss, Jena, Germany) with a 25× water immersion objective (LD LCI Plan-Apochromat 25×/0.8 Imm Korr DIC M27) or a Leica SP8 CLSM (Leica; Wetzlar, Germany) with a 40× water immersion objective (Leica; 1.1NA), respectively. DAPI, Alexa Fluor 488, 555, and 647 were excited with laser lines of 405 nm, 499 nm, 553 nm, and 653 nm (LSM710), respectively, or with a white light laser (SP8).

### 2.5. Statistical Analysis

Statistical analysis was performed using Graphpad Prism 5.04. After checking normal distribution of data with a Kolmogorov–Smirnov test, we performed nonparametric tests. For pairwise comparisons, a two-tailed Mann–Whitney U test was performed, whereas for three experimental groups, a Kruskal–Wallis test and subsequent Dunn’s multiple comparisons test as post hoc test was applied. The level of significance was set to *p* < 0.05. Data are presented as mean ± standard deviation (SD). For quantitative image analysis, the ImageJ (V 1.53c) cell counter plugin was used [36]; control embryos with less than 8 cells in the ICM were excluded from the analysis.

## 3. Results

### 3.1. NANOG-KO Has No Effect on Blastocyst Rate but Results in Reduced Total Cell Number

After selection with puromycin, we generated 57 single-cell clones and achieved a mutation rate of 54.4% including 8 homozygous mutations (14.0%). Two different cell clones with an identical homozygous insertion of a single nucleotide, which induces a frameshift mutation, were used for SCNT to produce embryos without NANOG (*NANOG*-KO). SCNT embryos from two different cell clones with no mutation from the same transfection experiment (NT Ctrl) and embryos produced by in vitro fertilization (IVP Ctrl) served as controls. There were no differences regarding blastocyst rates between embryos from all four cell clones. SCNT embryos from both *NANOG*-KO cell clones showed consistent alterations as described below. The group of NT Ctrl embryos derived from the two *NANOG*-intact cell clones was phenotypically homogeneous. To verify the absence of NANOG protein, *NANOG*-KO blastocysts (*n* = 9) were stained for NANOG using two different antibodies, and no positive cells were observed (Supplementary Figure S1A). There was no significant difference between *NANOG*-KO and Ctrl embryos regarding blastocyst rates. *NANOG*-KO embryos were able to expand but appeared to be smaller than NT Ctrl blastocysts (Supplementary Figure S1B), with significantly decreased diameters of day 8 *NANOG*-KO compared to NT Ctrl blastocysts ( Supplementary Figure S1C). We analyzed the total cell number and the number of ICM and TE cells using SOX2 and CDX2 as markers, respectively. We found a significant reduction in both lineages in *NANOG*-KO embryos, while no significant difference in the ratio of ICM to total cell number was seen, showing a proportionally normal distribution of cells to ICM and TE during the first lineage differentiation in the absence of NANOG (Table 1).

### 3.2. NANOG Is Dispensable for Expression of Pluripotency Factors and Hypoblast Markers

**Figure 1:**
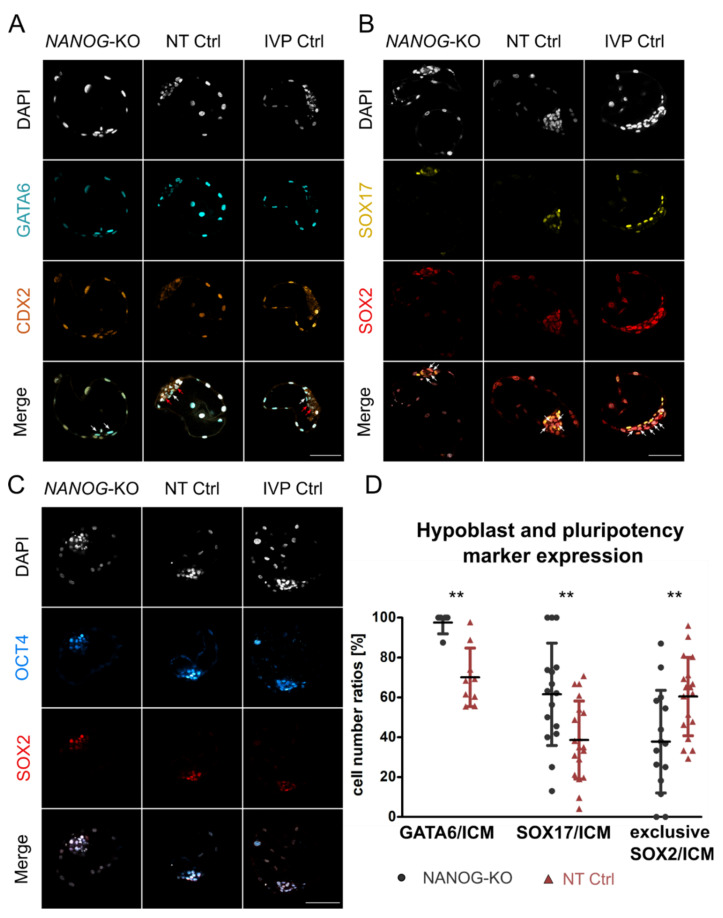
Expression of hypoblast and pluripotency markers in *NANOG*-KO and control groups. Representative confocal planes of day 8 blastocysts stained for GATA6/CDX2 (**A**) and SOX17/SOX2 (**B**). Sample sizes of GATA6/CDX2 were *n* = 5, 9, 14 and of SOX17/SOX2 *n* = 16, 18, 10 for *NANOG*-KO, NT Ctrl, and IVP Ctrl, respectively. White arrows indicate ICM cells with GATA6 expression (**A**) or exclusive SOX2 expression (**B**), red arrows indicate GATA6/CDX2 double negative cells (**A**) in the ICM. (**C**) Expression of pluripotency factors OCT4 and SOX2. Sample sizes were *n* = 4, 9, 4 for *NANOG*-KO, NT Ctrl, and IVP Ctrl, respectively. Color codes were: Grey (DAPI), cyan (GATA6), orange hot (CDX2), yellow (SOX17), red (SOX2), and cyan hot (OCT4). (**D**) The ratio of GATA6, SOX17, and SOX2 positive cells within the ICM of *NANOG*-KO (black) and NT Ctrl (red) embryos. SOX2 served as ICM marker in the quantification of SOX17. SOX2 exclusive expression represents cells positive for SOX2 while negative for SOX17. Data were analyzed using a two-tailed Mann–Whitney U test and are presented as mean (%) ± standard deviation. Asterisks (**) indicate significant differences between groups (*p* < 0.01). Sample sizes of GATA6 were *n* = 5, 9; of SOX17 *n* = 16, 19; and of SOX2 exclusive *n* = 15, 18 for *NANOG*-KO and NT Ctrl embryos, respectively. Scale bars indicate 100 µm.

We stained *NANOG*-KO, NT Ctrl, and IVP Ctrl day 8 blastocysts for GATA6/CDX2 (Figure 1A) and SOX17/SOX2 (Figure 1B). In both control groups, embryos showed consistent co-expression of CDX2 with GATA6, and a subset of the CDX2 negative ICM cells was also GATA6 negative. Staining of NT Ctrl (*n* = 9) and IVP Ctrl blastocysts (*n* = 18) for NANOG and GATA6 (Supplementary Figure S2A) confirmed that GATA6 negative cells express NANOG, resulting in the previously reported mutually exclusive expression of these lineage markers [16,18]. SOX2 was expressed in the entire ICM, and a subset of cells already expressed the late HB marker SOX17. In *NANOG*-KO day 8 blastocysts, an ICM was clearly discernible by CDX2 negative cells, while GATA6 was expressed ubiquitously with no negative cells in the ICM or TE.

The ratio of SOX17 positive cells within the ICM increased significantly compared to NT Ctrl (61.6% ± 25.7% vs. 38.6% ± 19.6%, respectively) but not IVP Ctrl (56.1% ± 13.5%), while cells with exclusive SOX2 expression were still present, albeit at reduced numbers (Figure 1D). We conclude that NANOG is required for the repression of GATA6 in the ICM. In contrast to mouse *Nanog*-KO embryos that show complete loss of SOX17 [2], we still found SOX17 positive cells in the ICM. However, absence of NANOG and a ubiquitous GATA6 expression is not sufficient to induce a pan-ICM expression of SOX17 in bovine blastocysts.

Staining for OCT4 and SOX2 showed that in NT Ctrl and IVP Ctrl embryos, both factors are co-expressed throughout the entire ICM and that in the absence of NANOG, this pattern is maintained (Figure 1C). None of the embryos showed OCT4 expression in the TE at day 8.

### 3.3. Inhibition of MEK Induces Cell Death in NANOG-KO Embryos

**Figure 2:**
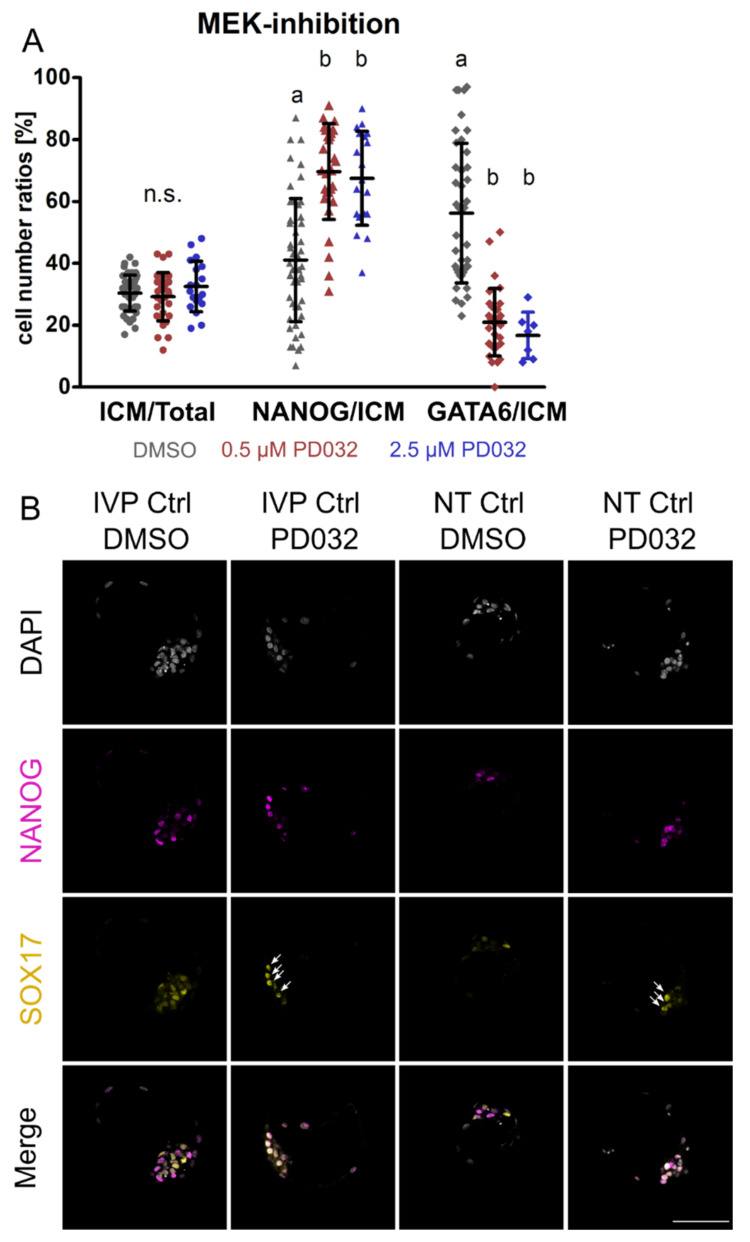
The effect of different dosages of MEK-inhibitor PD0325901 (PD032) on the expression of NANOG, GATA6, and SOX17. (**A**) The ratio of ICM/Total, NANOG/ICM, and GATA6/ICM in the presence of 0.5 and 2.5 μM PD032 in IVP Ctrl embryos. The proportion of ICM to total cell number (Total) is shown, and the number of NANOG and GATA6 expressing cells was set in relation to the number of ICM cells. Embryos were cultured from day 5 morula to day 8 blastocyst in the presence of 0.5 and 2.5 μM PD032. Data were analyzed by Kruskal–Wallis with Dunn’s multiple comparisons test as post hoc test and are presented as mean ± standard deviation. Different superscripts (a, b) indicate significant differences between groups (*p* < 0.0001), n.s. = not significant. Sample sizes of ICM/Total and NANOG/ICM were *n* = 49, 30, 19 and of GATA6/ICM *n* = 40, 30, 7 for DMSO (grey), 0.5 μM (red) and 2.5 μM (blue) PD032, respectively. (**B**) Representative confocal planes of NANOG and SOX17 expression in IVP Ctrl and NT Ctrl blastocysts cultured with DMSO or 2.5 μM PD032. Sample sizes of embryos stained for NANOG (magenta) and SOX17 (yellow) were *n* = 49 for IVP Ctrl DMSO, *n* = 19 for IVP Ctrl PD032, *n* = 8 for NT Ctrl DMSO, and *n* = 9 for NT Ctrl PD032. DAPI = grey; arrows indicate SOX17 expression in the presence of 2.5 μM PD032; scale bar indicates 100 µm.

In the next step, we aimed to investigate the effect of *NANOG*-KO while inhibiting the MEK signaling pathway. Because previous reports on the effect of the MEK 1/2 inhibitor PD032 in bovine embryos are in conflict [18,22], we first set out to test the effect of different dosages on the expression of NANOG and GATA6 in IVP Ctrl embryos. There was no difference between the DMSO control (*n* = 11) and the dosages 0.5 (*n* = 4) and 2.5 µM (*n* = 10) PD032 regarding the blastocyst per morula rate (45.6% ± 12.5%, 46.8% ± 6.1%, 50.0% ± 16.3%, respectively) and the ratio of ICM to total cell number (30.4% ± 5.8%, 29.2% ± 7.8%, 32.5% ± 8.2%, respectively). The number of ICM cells was determined without a specific staining on the basis of the embryos’ morphology. In agreement with Kuijk, et al. [18], the proportion of NANOG positive cells was markedly increased, while the expression of GATA6 was reduced but not completely switched off at both concentrations (Figure 2A). Similarly to GATA6, SOX17 was significantly reduced but still present at a concentration of 2.5 µM (Figure 2B). At 2.5 µM, both HB markers were always co-expressed with NANOG and thus failed to establish a mutually exclusive expression pattern.

As a higher dosage (2.5 µM) of PD032 did not affect blastocyst development or cell numbers, we performed inhibition of the MEK pathway in SCNT embryos using this concentration. We found similar blastocyst per morula rates (*p* > 0.05) of NT Ctrl in DMSO (*n* = 3; 52.4% ± 16.7%) and PD032 (*n* = 4; 42.5% ± 14.8%). The expression pattern of NT Ctrl embryos incubated with the MEK inhibitor was comparable to that of IVP Ctrl embryos that underwent the same treatment, as HB markers were still present (Figure 2B). Although treatment of *NANOG*-KO embryos with DMSO did not affect the blastocyst per morula rate (*n* = 3, 57.8% ± 16.2%) when compared to NT Ctrl treated with DMSO, incubating *NANOG*-KO embryos in the presence of PD032 (*n* = 5) resulted in severely compromised viability, and all embryos died. This agrees with findings in mouse embryos and mouse ESCs, where loss of NANOG and inhibition of MEK also result in cell death [2,26].

### 3.4. FGF4 in NANOG-KO Embryos Does Not Convert the Entire ICM to Hypoblast Precursor Cells

**Figure 3:**
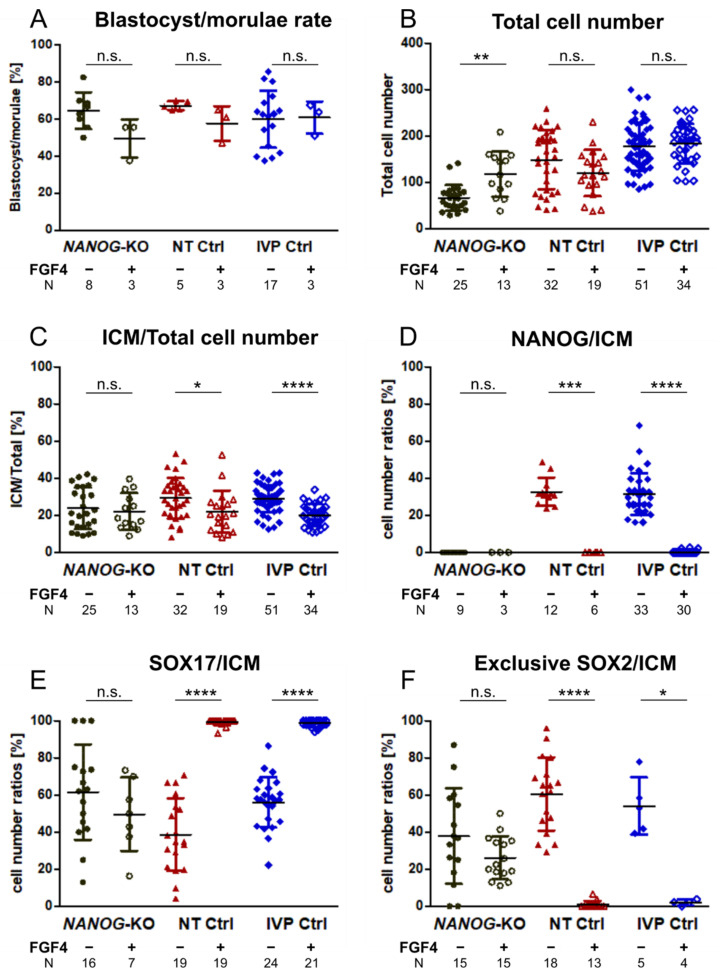
Developmental rates and cell number ratios of NANOG, SOX17, and exclusive SOX2 in *NANOG*-KO, NT Ctrl, and IVP Ctrl day 8 embryos treated with exogenous FGF4 and heparin. (**A**) Blastocyst per morula rate, (**B**) total cell number, (**C**) proportion of ICM to total cell number, (**D**) ratio of NANOG-positive cells in the ICM, (**E**) ratio of SOX17-positive cells in the ICM, and (**F**) ratio of cells exclusively expressing SOX2 in the ICM of *NANOG*-KO (black), NT Ctrl (red), and IVP Ctrl (blue) embryos without (−) and with (+) FGF4 and heparin are presented. ICM cells were determined by staining of SOX2. Data were analyzed by two-tailed Mann–Whitney U test and are presented as mean (%) ± standard deviation. Asterisks indicate significant effects of FGF4 treatment within embryo group. N = number of analyzed embryos, * *p* < 0.05; ** *p* < 0.01; *** *p* < 0.001; **** *p* < 0.0001; n.s. = not significant.

**Figure 4:**
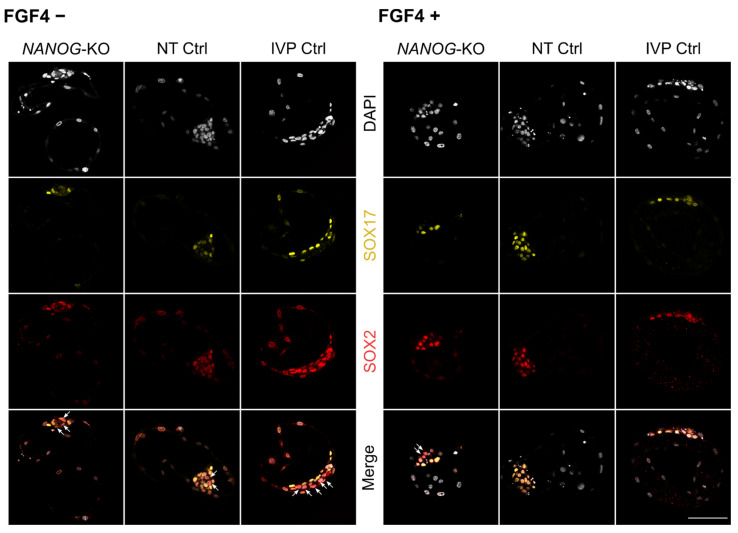
Expression of pluripotency and late hypoblast markers in *NANOG*-KO, NT Ctrl, and IVP Ctrl day 8 embryos treated with exogenous FGF4 and heparin. Representative confocal planes of day 8 blastocysts stained for SOX17/SOX2. Embryos without (−) and with (+) FGF4 and heparin treatment are presented. Arrows indicate ICM cells with exclusive SOX2 expression (SOX17 negative). Color codes are: Grey (DAPI), yellow (SOX17) and red (SOX2). Scale bar indicates 100 µm.

Subsequently, we investigated whether exogenous FGF4 can induce full SOX17 expression in *NANOG*-KO bovine embryos. In IVP Ctrl and NT Ctrl embryos, treatment with FGF4 completely switched off the expression of NANOG (Figure 3D), and most ICM cells expressed SOX17 (Figure 3E, Supplementary Figure S2B). FGF4 had no effect regarding blastocyst per morula rate and total cell number, while the ratio of SOX2 positive cells, i.e., the ICM, to total cell number was significantly reduced in both groups (Figure 3A–C). Treatment of *NANOG*-KO embryos with FGF4 did not affect the blastocyst per morula rate or the ICM to total cell number ratio, but the total cell number increased significantly with *NANOG*-KO embryos (117.6 ± 48.7 vs. 66.4 ± 27.3 without FGF4 treatment) reaching a total cell number similar to that of untreated NT Ctrl (148.6 ± 65.6, Table 1). In all embryos treated with FGF4, the ubiquitous expression of SOX2 in the ICM was maintained (Figure 4). As the SOX17 expression increased in FGF4 treated Ctrl groups, the exclusive expression of SOX2 was significantly reduced, whereas in mutant embryos, SOX2 exclusive expression remained unchanged (Figure 3F, Figure 4). The percentage of SOX17 positive cells in the ICM did not increase in *NANOG*-KO embryos (Figure 3E), which is in contrast to mouse *Nanog*-KO embryos, where exogenous FGF4 induces SOX17 expression in most of the ICM cells [2]. We conclude that in bovine, NANOG is required for FGF4 mediated expression of SOX17, as FGF4 alone was not sufficient to convert all ICM cells to SOX17 expressing HB precursor cells.

## 4. Discussion

To investigate the regulation of differentiation and maintenance of pluripotency during mammalian preimplantation development, it is vital to examine models other than mouse, as species-specific differences exist. The bovine embryo is a very suitable alternative, as IVP procedures are highly developed and similarities to human embryo development have been reported (reviewed in [23]).

In this study, we focused on NANOG, because it is not clear whether the role of this pluripotency factor during the second lineage segregation is conserved between mammals. In order to achieve uniform modification of all cells of the embryo, we used SCNT to produce *NANOG*-KO embryos instead of zygote injection, where mosaicism may hamper analyses. To exclude the effects of the SCNT procedure on the phenotype, we implemented two control groups: SCNT embryos generated from transfected cells that maintained the wildtype genotype (NT Ctrl) and embryos from in vitro fertilization (IVP Ctrl). NT Ctrl did not vary from IVP Ctrl embryos in any of the examined parameters, except for the proportion of SOX17 cells in the ICM, which was decreased in NT Ctrl embryos. We set the number of cells expressing lineage marker proteins in relation to the number of ICM cells in order to account for variations due to the different sizes of the embryos, especially the reduced size of *NANOG*-KO embryos. Staining embryos with markers for TE and ICM, i.e., CDX2 and SOX2, respectively, enabled us to quantify reliably the cell numbers in each lineage after the first differentiation. We found that the ratio of ICM to total cell number did not change in *NANOG*-KO embryos, showing that NANOG is not required for proper segregation of TE and ICM, as reported in mouse. On the other hand, we found a significant reduction in total cell numbers, which is in contrast to mouse *Nanog*-KO embryos, where the loss of NANOG does not impede cell proliferation until the E3.5 blastocyst stage [1]. Interestingly, the reduced total cell number in *NANOG*-KO embryos reached normal levels when the embryos were cultivated with exogenous FGF4, where the proliferative impact of FGF4 [17,37,38,39] evidently alleviated the reduction of total cell numbers in the absence of NANOG. We hypothesize that in *NANOG*-KO embryos, the absence of FGF4 expressing EPI precursor cells causes the reduced cell number. This suggests that EPI cells express FGF4, which to our knowledge has not been shown yet in bovine but is known in mouse [1].

Although Ortega, et al. [30] found a reduction of *SOX2* transcripts in bovine *NANOG*-KO embryos, we detected SOX2 and OCT4 expression in the absence of NANOG on the protein level. To our knowledge, this is the first report on SOX2 expression in the absence of NANOG in a mammalian embryo. We were not able to detect OCT4 in the TE of day 8 blastocysts using a monoclonal antibody, which is in contrast to Berg, et al. [40] and Simmet, et al. [11], who detected OCT4 in the TE of ex vivo day 11 or in vitro day 7 blastocysts using a different polyclonal antibody, respectively.

In bovine *NANOG*-KO embryos, the ICM ubiquitously expresses the early HB marker GATA6, which agrees with previous reports on bovine and mouse NANOG deficient embryos [2,30]. Interestingly, in bovine *NANOG*-KO embryos, expression of the later HB marker SOX17 was still present, but the absence of NANOG and a ubiquitous GATA6 expression was not sufficient to induce a pan-ICM expression of SOX17, as some cells in the ICM still showed exclusive SOX2 expression, making the regulation of SOX17 in the bovine embryo partly independent of NANOG and GATA6.

This is in sharp contrast to the mouse *Nanog*-KO, where expression of the late PE markers GATA4 and SOX17 completely fails but can be rescued in a chimeric complementation assay or fully induced by exogenous FGF4 [1,2,25].

We further investigated the second lineage segregation in bovine blastocysts by inhibiting the MEK pathway with PD032. In line with previous reports, MEK-inhibition did not completely ablate GATA6 positive cells [18,20], and also SOX17 was still expressed in the ICM. Canizo, et al. [22] found a dosage-dependent effect of PD032 with the concentration also applied in this study abolishing all SOX17. The reasons for these contrasting results remain unclear, and we can only speculate that the different embryo culture media have an effect on SOX17 expression in the presence of PD032. Bovine embryos were cultured in PD032 concentrations of up to 100 µM, and reduction of *SOX17* transcripts was already achieved at 10 µM, while higher dosages did not further decrease transcript abundance [19]. Treating bovine embryos with a broad-spectrum inhibitor of receptor tyrosine kinases (RTKs) including MEK (BI-BF1120) increased the abundance of *SOX17* transcripts, suggesting that SOX17 does not depend on direct activation via the MEK pathway [41]. We further found that HB markers were generally co-expressed with NANOG when the MEK pathway was blocked. Therefore, we suggest that NANOG mediated repression of HB markers is dependent on MEK signaling. Our data and previous reports indicate that, in bovine embryos, GATA6 and SOX17 are partly independent of the MEK pathway and that a so far unknown factor plays an important role in the regulation of HB differentiation.

When combining inhibition of MEK with loss of NANOG, we found that the viability of those embryos was severely compromised, resulting in cell death. Similar reports exist in NANOG deficient mouse embryos and ESCs, where cell death is observed after adding inhibitors of the MEK-pathway [2,26]. We conclude that HB formation, i.e., expression of GATA6 and SOX17, in the absence of both NANOG and a functioning MEK pathway is associated with cell death. We speculate that apoptosis is induced during the cell sorting process to eliminate cells that do not commit to either EPI or HB, as selective apoptosis was described for appropriate segregation of PE and EPI in mouse blastocysts [5,42]. Nevertheless, our hypothesis cannot explain why the TE is also affected by cell death.

In the mouse, the loss of FGF4 expressing EPI precursor cells leads to complete ablation of late PE marker expressing cells that can be rescued with exogenous FGF4 [2,43]—evidence that FGF4 alone is sufficient to induce PE differentiation. The regulation of SOX17 expression in bovine embryos appears to be different, as FGF4 alone without functional NANOG was not sufficient to convert all ICM cells to SOX17 expressing HB precursor cells. Thus, we conclude that NANOG is required for FGF4 mediated expression of SOX17.

Our results show that in the bovine embryo, the establishment of HB precursor cells is independent of EPI-cell mediated FGF/MEK signaling. This is in sharp contrast to mouse but similar to human, where the FGF/MEK pathway does not regulate the second lineage differentiation [18,21]. An unknown factor induces HB differentiation, and it is of utmost interest to further investigate this pathway and whether it also exists in human embryos as well.

## Figures and Tables

**Table 1:** Developmental rates and cell numbers of day 8 *NANOG*-knockout (*NANOG*-KO), nuclear transfer control (NT Ctrl), and in vitro-produced control (IVP Ctrl) embryos. Data are presented as mean ± standard deviation. Different superscript letters (a, b) within a row indicate significant differences (*p* < 0.05). Data were analyzed by Kruskal–Wallis test with Dunn’s multiple comparisons test as post hoc test.

Experimental Group	*NANOG*-KO	NT Ctrl	IVP Ctrl
No. of experiments	8	5	17
Blastocysts/zygotes [%]	29.0 ± 12.9	36.1 ± 9.9	28.3 ± 4.2
No. of analyzed embryos	25	32	51
Total cell number	66.4 ± 27.3 ^a^	148.6 ± 65.6 ^b^	177.4 ± 52.2 ^b^
ICM/total cell number [%]	23.8 ± 11.3	29.2 ± 11.0	29.0 ± 7.3
ICM number	15.8 ± 10.5 ^a^	43.5 ± 24.5 ^b^	51.3 ± 20.0 ^b^
TE cell number	50.6 ± 22.6 ^a^	105.1 ± 47.2 ^b^	126.1 ± 40.5 ^b^

## Data Availability

Not applicable.

## Supplementary Materials

The following are available online at www.mdpi.com/article/10.3390/cells10092232/s1, Figure S1: Producing bovine *NANOG*-KO embryos via somatic cell nuclear transfer (SCNT); Figure S2: Expression of epiblast and hypoblast markers in NT Ctrl and IVP Ctrl day 8 embryos; Table S1: Targets, antibodies, suppliers, and applied dilutions for immunofluorescence staining.

## Abbreviations

BME	Basal Medium Eagle’s amino acids solution
CLSM	Confocal laser scanning microscope
EPI	Epiblast
ESCs	Embryonic stem cells
FGFR	FGF-receptor
HB	Hypoblast
ICM	inner cell mass
IVP	In vitro production
KO	Knockout
MAPK	Mitogen-activated protein kinase
MEM	Minimum Essential Medium
OCS	Estrous cow serum
PD032	PD0325901
PE	Primitive endoderm
RTK	Receptor tyrosine kinase
SCNT	Somatic cell nuclear transfer
SD	Standard deviation
TE	Trophectoderm
