# Supplementary material for: Hypoblast Formation in Bovine Embryos Does Not Depend on NANOG
Source: Cells. 2021 Aug 28;10(9):2232. doi: 10.3390/cells10092232 (PMC8466907; doi:10.3390/cells10092232)
Supplement: Supplementary file 1 [file cells-10-02232-s001.zip › cells-1321834-supplementary.pdf]

Supplements

1. Figure S1

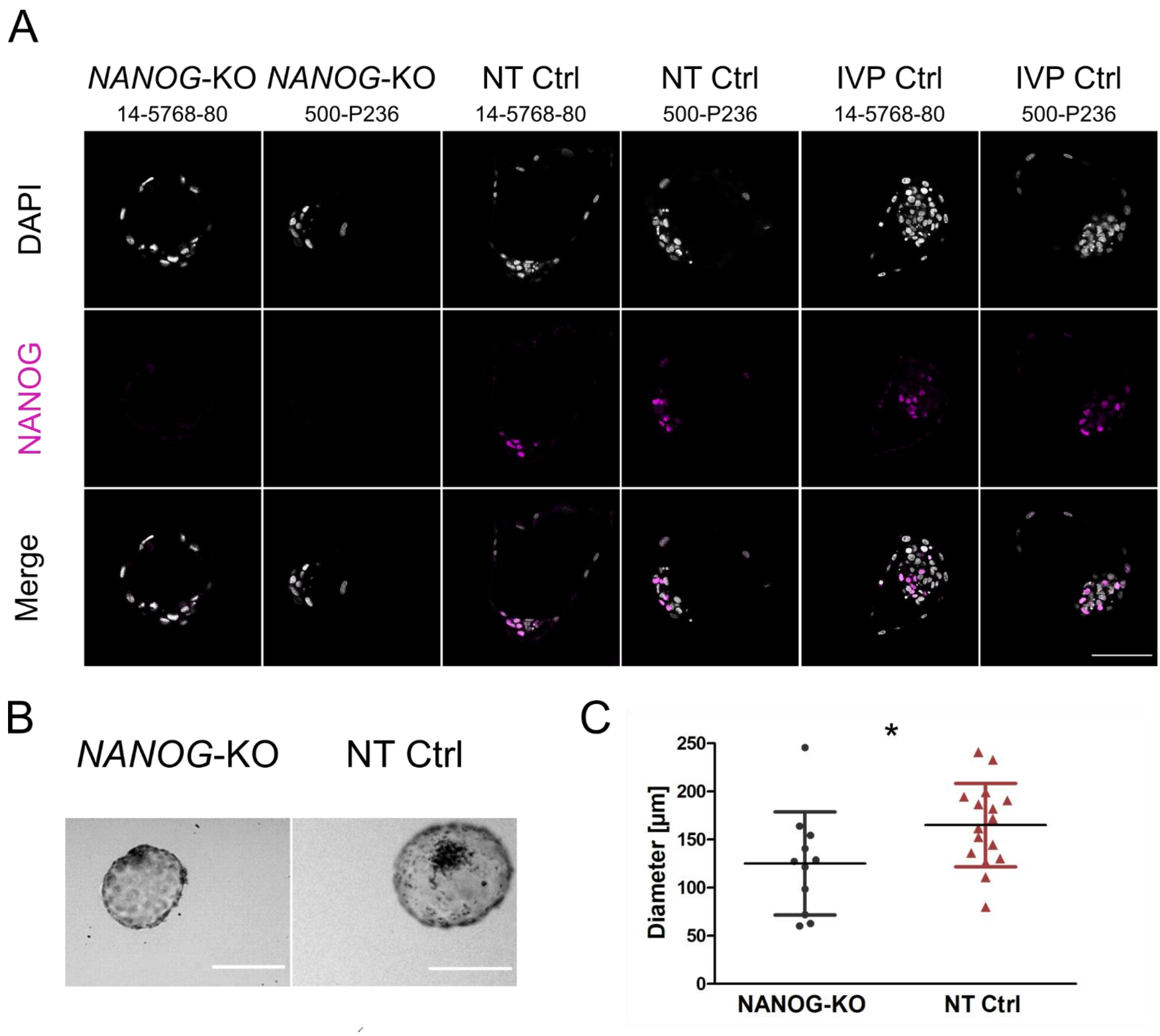

Figure S1. Producing bovine *NANOG*-KO embryos via somatic cell nuclear transfer (SCNT). **(A)** Representative confocal planes of day 8 *NANOG*-KO and control blastocysts from somatic cell nuclear transfer (NT Ctrl) and in vitro fertilization (IVP Ctrl) stained for NANOG with two different primary antibodies (Table S1). **(B)** Brightfield images of *NANOG*-KO and NT Ctrl day 8 blastocysts. **(C)** Mean diameter ± standard deviation of *NANOG*-KO (n=11) and NT Ctrl (n=16) day 8 blastocysts. Data were analyzed by two-tailed Mann-Whitney U test (* $p < 0.05$). All scale bars indicate 100 µm.

2. Figure S2

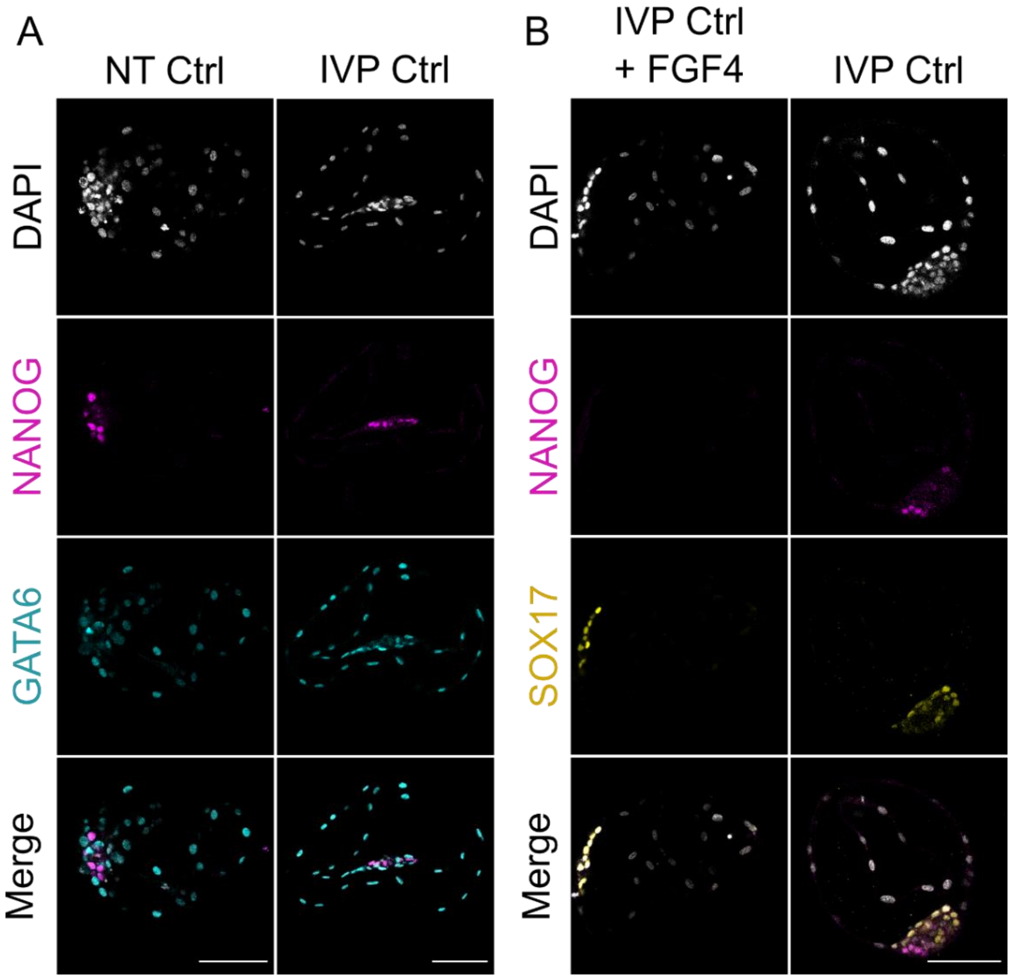

Figure S2. Expression of epiblast and hypoblast markers in NT Ctrl and IVP Ctrl day 8 embryos. **(A)** Representative confocal planes of day 8 NT Ctrl and IVP Ctrl blastocysts stained for NANOG and GATA6. (**B**) IVP Ctrl embryos were cultured from morula to day 8 blastocyst in the presence of FGF4 and heparin (each 1 µg/ml) and stained for NANOG and SOX17. Scale bars indicate 100 µm.

3. Table S1

Table S1. Targets, antibodies, suppliers and applied dilutions for immunofluorescence staining. Ig = immunoglobulin.

**Target**	**Antibody**	**Supplier**	**Dilution**
NANOG_01	Rabbit anti-human NANOG (500-P236)	Peprotech	1:600
NANOG_02	Mouse anti-human NANOG (14-5768-80)	Thermo Fisher	1:250
GATA6	Goat anti-human GATA6 (AF1700)	R&D Systems	1:500
SOX17	Goat anti-human SOX17 (AF1924)	R&D Systems	1:100
SOX2_01	Goat anti-human SOX2 AF2018	R&D Systems	1:500
SOX2_02	Rabbit anti-human SOX2 (AB5603)	Millipore	1:1000
OCT4	Rabbit anti-human OCT4 monoclonal ab181557	Abcam	1:250
CDX2	Rabbit anti-human CDX22 ab88129	Abcam	1:250
Rabbit Ig	Donkey anti-rabbit Alexa Fluor 555 (ab150074)	Abcam	For NANOG_01: 1: 800<br>For OCT4: 1:800<br>For SOX2_02: 1:1000
Rabbit Ig	Donkey anti-rabbit 711-605-152 Alexa 647	Jackson Immuno Research	For CDX2: 1:400
Mouse Ig	Donkey anti-mouse Alexa Fluor 647 (715-605-150)	Jackson Immuno Research	For NANOG_02: 1:400
Goat Ig	Donkey anti-goat Alexa Fluor 633 (A212082)	Thermo Fisher	For GATA6: 1:400
Goat Ig	Bovine anti-goat Alexa Fluor 488 (805-545-180	Jackson Immuno Research	For GATA6: 1:1000<br>For SOX17: 1:200<br>For SOX2_01: 1:500
